# Night nursing – staff's working experiences

**DOI:** 10.1186/1472-6955-7-13

**Published:** 2008-10-31

**Authors:** Kerstin Nilsson, Ann-Mari Campbell, Ewa Pilhammar Andersson

**Affiliations:** 1 University of Gothenburg, the Sahlgrenska Academy, Institute of Health and Care Sciences, Göteborg, Sweden; 2 University of Skövde, School of Life Science, Skövde, Sweden; 3 Malmö University, Faculty of Health and Society, Malmö, Sweden

## Abstract

**Background:**

Although the duties and working conditions of registered, and enrolled nurses have previously been described from different perspectives, they have not been examined from the night nursing aspect. The aim of the study was to describe the night nursing staff's working experiences.

**Methods:**

The design of the study is qualitative and descriptive. Interviews were conducted with 10 registered and 10 enrolled nurses working as night staff at a Swedish University Hospital. The interview guide was thematic and concerned the content of their tasks, as well as the working conditions that constitute night nursing. In addition, the interviews were transcribed verbatim and analyzed using content analysis.

**Results:**

The night duties have to be performed under difficult conditions that include working silently in dimmed lighting, and making decisions when fatigue threatens. According to the night staff, its main goals are to provide the patients with rest and simultaneously ensure qualified care. Furthermore, the night nursing staff must prepare the ward for the daytime activities.

**Conclusion:**

The most important point is the team work, which developed between the registered and enrolled nurses and how necessary this team work is when working at night. In order for nurses working at night to be fully appreciated, the communication between day and night staff in health care organizations needs to be developed. Furthermore, it is important to give the night staff opportunities to use its whole field of competence.

## Background

In the main, intrinsic value is not attached to night work which tends to make night nursing invisible. Night work is apprehended as separated from day work and night work sometimes seems to be less valuable [1]. Night nursing is described as being controlled by plans made in the daytime and staffed with fewer nurses on the wards [2,3]. Therefore, this study focuses on how the two occupational groups working at the hospital wards at night in Sweden, registered nurses (RNs) and enrolled nurses (ENs), experience their work at night.

All professionals in Swedish health care organizations are responsible for their own actions. The general rule is that the one who performs health care tasks shall have necessary competence in order to guarantee good and secure health care [4]. RNs are principally responsible for nursing and ENs are principally responsible for caring. RN are allowed to delegate nursing tasks to an EN if the EN has real (not formal) competence fore the task [5]. Thereby RNs and ENs mostly complete each other and sometimes ENs can be seen as RNs' assistant.

RNs are expected to be skilled in different areas including both caring and nursing. The nursing discipline could be regarded as a practical one which joins knowledge from the fields of both the Human, and Biomedicine Sciences. According to Kim, nursing includes four domain typologies: the client, the client-nurse, the practice, and the environment domains [6,7]. The employers' association and trade unions, together with The Society of Nursing and the Board of Health and Welfare, have formulated a description of the competence needed. RNs are required to examine and treat patients independently, or in cooperation with physicians, and to carry out medical prescription. Furthermore, the promotion of good health, the prevention of ill health, as well as the development of safe and secure care, are also fields demanding nursing competence. In addition, RNs are expected to be skilled in nursing development, which means to have the ability to independently and continuous analysis strengthens and weakness in own professional competence. They are also expected to stand up for personal and professionall development of nursing competence, as well as in teaching both patients and staff. They are also expected to lead, prioritize, distribute and coordinate nursing care, as well as cooperate with other professions [8]. However, this is a general competence description and does not provide any guide for the skills required in night nursing. Previously, the Board of Health and Welfare declared that a RN with continuous night work ought to have the possibility of developing professional nursing skills and obtaining nursing experience [9]. In the descriptions of nursing competence required from the National Board of Health and Welfare in 2005 [8], there are no distinctions between day and night work, or between RNs and ENs. In fact, Sweden has not developed a national description of the competences required of ENs. It is worth mentioning that Swedish ENs have a three year upper secondary school education. At present, a three-year university education leading to a Bachelor's degree is compulsory to become a RN in Sweden.

The duties of nurses in relation to specific situations or patients with special diagnoses have been studied previously. Such examples include a duty to prevent a patient from self-inflicted injuries [10], military nursing in a combat environment [11], strategies providing care for women with post partum psychosis [12], or the duty of attempting to identify and meet children's need of spiritual care [13]. A main nursing duty in the operating room is to secure patient safety and prevent mistakes [14]. In an acute setting, the nurses' hospital-based practice of health promotion mainly consists of providing prescriptive and individual preparatory information including for example encouragement, explanations and instructions [15].

In a study of the work content of nurses at a Swedish hospital, it was revealed that 38% of their working time consisted of direct nursing care of patients, while the remaining time was used for other work-related tasks [16]. Nurses found that their professional roles are vague, because the distinction between ENs and RNs is unclear. However, the roles were also described as being that of 'a spider in the web', which implied being coordinator, controller and leader [17]. In addition, previous studies have examined the working conditions of nurses in relation to stress [18,19], their experiences of work satisfaction [20-22] and downsizing [23-27].

When the literature review focused on the duties and working conditions of night nursing, fewer studies were found. Nevertheless, in a study about how learning at night was accomplished, it was found that the monitoring and assessment of the patients were of utmost importance for the provision of good and safe nursing care at night [28]. Furthermore, in a study which measured the time spent for direct and indirect care during three duty-shifts, it was found that the evening shift used most of its time on direct nursing activities, while the night shift spent most of its time on indirect nursing tasks [29]. When an instrument was used to evaluate the nursing care provided at night, it was found that night nurses need to improve their ability to assess the care needs of patients at night [30].

Other existing studies in relation to night nursing mainly concern problems associated with changing the 24-hour body rhythm [31-34], difficulties with the working environment [2,35], the health status of shift-workers [36-38], as well as staying awake while driving home after a night shift [39]. Furthermore, some studies describe the difficulties of patients being able to sleep at night undisturbed [40], while others compare night nurses' and patients' satisfaction with nursing care at night [30]. The work of the night staff has been found to be largely invisible and unknown [41], and there are still few studies which describe the duties of night nurses. Therefore, the aim of this study was to describe the night nursing staff's working experiences. The study focused on the following issues: what constitutes the night work of the RNs and the ENs, and under what kinds of working conditions does the night nursing staff perform its duties?

## Methods

A descriptive, qualitative research design was selected, with the aim of contributing new insights into the work of RNs and ENs at night. This design makes it possible to obtain knowledge and understanding about the meaning people give to actions, processes, beliefs and values in naturalistic settings [42]. Qualitative design has been found appropriate when research and/or literature on the phenomenon of interest is limited [43]. Based on the aim of the study, the open-ended interviews were chosen as the method of data collection. A thematic interview guide (see appendix) was used to guide the interviews. In the interview situation, the researcher tries to understand the interviewee's perspective and experiences. The results of the interviews depend on the quality of the interviewer-interviewee interaction, as well as on culturally inherent assumptions about how experiences, feelings and intentions are understood [44]. It is the outcome of the interviews that is analyzed and not the interaction [45].

### Procedure

The heads of four departments, (Intensive Care Unit, Medicine, Surgery and Geriatrics/Rehabilitation, divided into eight wards), at a Swedish University Hospital were asked if they would like to participate in this study. These four departments (including eight wards) were chosen from six at the hospital, because they all provided 24 hour care and had recently undergone organizational changes. All four department heads gave their permission for the study to be carried out.

From a total of 22 RNs and 21 ENs, working as permanent night staff on the eight wards, 11 RNs and 11 ENs were purposefully [46] selected for the study to obtain a variation in age and experience as this vouched for richness in data, i.e. descriptions of working at night. The purposeful selection was guided by the information of professional role, age and year of employment given in the respective staff lists of each ward. For the selection, all the wards were regarded as one entity. The above criteria were used for the selection process [47]. Two of the selected nurses did not wish to participate.

The interviews were conducted according to an interview guide (see appendix in additional file 1) comprising the following themes: the content of work, task changes over time, and the working conditions that constitute night nursing duties. The questions were followed with more penetrating enquiries, and with questions such as: can you describe that in more detail, and can you explain? The interview guide was developed in relation to the study questions and the researchers' experience from the field in question and was not changed during the period of data collection. All the interviews were tape-recorded and transcribed verbatim. Each interview lasted from 45 to 120 minutes. Fifteen of the interviews were conducted at the workplace, 12 at the beginning of the shift and three during the day, while four were carried out in the participants' homes, and one in a neutral place.

### Analysis

The interviews were analyzed using manifest content analysis that means the analysis has focused on what the text said, i.e. visible and obvious content [44]. After the interviews were transcribed, they were read through to gain a sense of the whole. The unit of analysis was the interview text in its entirety [48]. Units with a meaningful relation to the aim of the study were then identified. These units of meaning consisted of words, sentences and text sequences and emerged from the data. The meaning units were labeled with a code. The codes were related to the comprehensive content of the meaning units. The phase of condensation or abstraction of the meaning units was omitted, since the discovered units of meaning were sufficiently condensed and manageable in their original forms. The differences and similarities in the codes were then compared and sorted into content categories. Finally, themes emerged through reflecting about the categorized content.

### Demographic information

Twenty participants, of which 19 were female and one was male, were interviewed. The median age was 45 years, and their ages ranged from 31–60 years. The average length of night work experience was 11.9 years for the RNs and 13.2 years for the ENs, and they only worked at night. (Table 1)

**Table 1:** Participants in the study

**Division**	**RN**	**EN**
ICU	2	3
Medicine	3	1
Surgery	2	2
Geriatrics/Rehabilitation	3	4

***Total***	***10***	***10***

### Ethical considerations

According to Swedish law, no approval from an ethics committee was required for this type of study at the time it was conducted. Permission to carry out the study was provided by the head of each department. Furthermore, informed consent was obtained from all the participants in accordance with the Declaration of Helsinki [49]. This means that the participants were informed about the purpose of the study, data collection method, their voluntary participation, the confidential treatment of data and that they could withdraw at any time. The information was given twice; the first time at the first telephone call and secondly at the interview occasion. To protect the participants' confidentiality, the interviews were coded with a number.

## Results

The results are presented in three themes: conceptions of night work, working conditions, and duties.

### Conceptions of night work

The conceptions of night work are related to what the night staff hears from the day staff. According to the night staff, these conceptions are based on a lack of knowledge about night work among the day time staff.

#### Holding the fort

The statements illustrate that it is difficult to describe what night work entails, and that it is not understood by the day nurses until they experience it. The interviewees revealed the tension existing between day and night staff.

Most of the day staff doesn't understand what we are actually doing at night, but it's this stuff about we only hold the fort, and it's not like that because it's awfully hard work between five and seven. At five we start washing our patients, and weighing and changing beds and the 24 hours ends at six, and then all drains and all pumps everything has to be read, and measured. We carry a very heavy burden between five and seven, but I don't really think that the day staff understands what we're doing, so we never get any real understanding for how much we really do (I 3).

#### Working at night gives days off

The lack of understanding about night work also leads to a lack of understanding of the night staff's need of rest during the day. The informants claim they need as much sleep, and under similar conditions, as the day staff.

... but many of them [day staff] say well it's very good to work nights because then you can lie and sleep on the beach in the summer, but one knows that doesn't work... I certainly can't cope with that, you know, so it's wearing (I 3).

#### The staff also sleeps at night

The patients also have conceptions based on the assumption that everybody sleeps at night. In such a circumstance, asking for help is equivalent to disturbing the night sleep of the night staff.

All the elderly people think that the night staff sleeps and they don't want to be a nuisance. We have to explain ... but we don't sleep, we are here at our job, we are awake at night and sleep in the daytime. Then they say, oh dear, oh dear, poor you, but then we tell them it's our own choice (I 20).

According to the participants, the perceptions are changing towards a better understanding of night work. One reason is that there is often a lack of night staff, which means that day staff has to work at night. The day nurses then become aware of what night work entails. Another reason is related to changes in the organization, where the heads of the ward play a decisive role in creating a sense of comradeship among the staff.

### Working conditions

The working situation at night is described as specific, because all activities on the ward are carried out in a subdued environment. This means that the tasks are performed under conditions which benefit the patients but are more difficult for the staff to carry out than during the daytime.

#### Working in silence

It has to be quiet and peaceful at night to enable the patients to sleep. When the fans are turned off, it becomes so quiet that all sounds are amplified and an ordinary conversation in the corridor can disturb the patients' night sleep. Consequently, the nurses lower their voices, and noisy duties, such as showering and working in the sluice, are avoided.

I'm very careful to tiptoe around, plus that you don't talk when you're inside the room if you don't need to (I 8).

Nevertheless, the ambition to create a silent night milieu can be difficult to achieve. Many elderly patients have poor hearing and when the nurses whisper, a patient can loudly ask what was said. This can consequently wake all the patients in the room, which means that the nurses' efforts to work quietly have been wasted. Another way to avoid disturbances at night is not to wheel newly admitted patients into the rooms. They are placed instead in a treatment room, day room or corridor until the morning work starts. A further way to muffle sounds is to wrap hand towels around door handles to prevent them from making a noise when opening and shutting the doors.

#### Working in dim light

In the evening, the lights on the ward are dimmed, and those in the rooms are switched off. When the patient's status, infusions or bandages are checked, this is done with the help of a torch, which means that the inspection is restricted to within the range of its beam.

Int: Do the patients look different in dim light?

I:16: Yes they do – definitely – you have to look more at, what shall I say, you listen more to their breathing when the patient is asleep, if it changes in any way, or some other disturbing element in the patient, maybe more often, and then you have to, because there's no light you might not see any variation in color, but you may–you feel.

Int: You feel and you listen?

I:16: Yes, exactly. To be able to notice if there is any change of any kind that you might have seen, that you perhaps see more in the daytime than, well so there are other senses.

#### Coping with fatigue

After the second night round (2 a.m.) has been carried out, fatigue starts to be trying and is felt creeping into the body. The RNs' statements revealed that it is at this point in time that medicines are dealt out and drugs are requisitioned. Concentration is low, and the work is tedious, because the tablets must be checked several times.

... you probably have to be a special person to have the strength to work at night, because the thing is that it's also a question of whether you, as an individual, can manage to keep awake a whole night, and I have seen workmates who are not very quick-witted after one o'clock at night, but are tired and exhausted (I 19).

#### Not being afraid of the dark

Working at night also requires that nurses have to leave the ward to attend to errands throughout the hospital alone. For example, they have to deliver samples to the laboratories, accompany patients to x-ray examinations, or transport to and from the operating theatre.

... dare go off on your own on a bicycle in the middle of the night all through the hospital, if you have to go off to the lab, or.and not be afraid of the dark /.../ because it's very empty you know, and it's locked everywhere (I 12).

If more than one member of staff is required to carry out a task outside the ward, another ward often has to stand by with its staff. When manpower is low, it is impossible for two nurses from the same ward to leave it at the same time.

#### Cooperation

A special relationship develops between RNs and ENs during night work, because this is when both categories of staff are dependent on each other. This dependence applies to both personal relations and professional knowledge.

Well, we know each other /.../ we depend on each other quite simply, and even the RNs themselves say that they think it's really nice (I 9).

How the work was divided between the two professional groups varied according to the composition of the team, but there were clear boundaries between the RNs' duties, which comprised handling medicines and making decisions about medication, and those of the ENs.

### Duties

As in the daytime, the work at night comprises general care, specific care and different forms of service tasks.

#### General care

The aim of general care at night is to provide the patients with good sleep to enable them to have the energy to cope with daytime treatment and training. In addition to distributing hypnotics/sleeping pills, the following types of care are applied: ensuring well-being, relieving anxiety and creating calmness.

Caring for the patients at night is an art; one almost has to treat patients with kid gloves (I 4).

Ensuring well-being implies ensuring that the patient lies comfortably in a dry and smooth bed, and that pillows are placed to relieve pressure and help the body relax. In addition, administering an analgesic, particularly before the painful process of being turned, also increases the feeling of well-being.

Patient anxiety is pronounced at night. This can occur after the day's activities and movements have quieten down, and the patients are left with their own thoughts and anxieties about their condition, treatment and what the future will bring.

During the night the demons and dreadful thoughts come, say the patients, and they become anxious and sad and start thinking why has this happened to me...and you have to listen and take your time (I 19).

Relieving anxiety is a theme that recurred in all the interviews, and the informants exemplified the different types of care provided to alleviate this problem. Nurses tried to reduce anxiety by sitting beside patients, holding their hands, being close by ready to answer a patient's summons, and taking the time, even if only for a few minutes. These kinds of measures can be of importance to the patient at night, and they all exemplify the care activities of the ENs and RNs. Another anxiety that many patients, particularly elderly ones, experience is physical in nature. Using the bathroom at night is not the same as during the day. Many patients who manage to visit the lavatory perfectly well in the daytime can have difficulties at night. They can be affected by the dimmed lighting, sleeping pills, fatigue, and a sense of disorientation about where they are and in what physical state they are in. All or any of these effects can lead to them wandering about and falling over.

#### Specific care

Organizational and structural changes at the hospital studied have reduced the differences between night and day work. Patients are now admitted at night, not only in emergency cases, but also from the waiting list. This means that specific measures to prepare patients for various treatments and interventions, such as x-ray examinations and laboratory tests, are frequently undertaken at night.

... and maybe we are going to take some patient's ECG and sometimes it is laboratory tests that have to be taken (I 11).

The RN is usually the only one responsible for assessing the patient's status and deciding whether and when a doctor should be called. As a result of the shortage of staff, irrespective of category, a system of relay doctors is used, that is, doctors who are employed to cover the vacancies that arise. These doctors seldom know the patient, which means the RN must, firstly, make comprehensive assessments, and, secondly, decide what information the doctor needs in order to determine the measures required. This nurse-doctor conversation is usually conducted on the telephone.

#### Service tasks

Changes in the duties have occurred gradually over the years. More tasks have been allocated to the night staff, for example, washing wheelchairs and beds, dusting, watering plants, preparing the breakfast, as well as laying the table, which are all felt to be a burden. The following statement illustrates the feeling these kinds of service tasks arouse:

... in some situations, I could be replaced by both a cleaning woman and a servant (I 18).

Other tasks that are done to help the day staff can include filling the wagons, sorting the washing and replenishing the stores. While such tasks are not obligatory, they are carried out in order to help, and out of a sense of duty and pride in being able to hand over a ward in excellent order to the day staff.

## Discussion

In this study, the experiences of the interviewed RNs and ENs varied, but taken together these experiences are extensive and also cover different health care units. This variation and range of experiences must be regarded a strength of the study, since they provide an understanding of the content of care. The interview situations were characterized by openness, as well as the nurses' willingness and interest in talking about their experiences, which facilitated understanding the interviewees. On the other hand people in the interview situation can choose to say what they want or feel to say at the moment, which could be seen as a limitation of the interview method [44]. Therefore attempting was made to control the interviews in order to gain answers to the questions still with maintenance of openness.

Consequently, the content of the interviews is rich. The interviewer has personal experience working as a nurse, both on day and night shifts, and was thus able to better understand the nurses' situation, which facilitated the interview situation considerably. This could also be seen as a weakness of the study, because some information could have been taken for granted and not penetrated further. In addition, the analysis may have been affected by the researcher's prior understanding of the work and competence needs of RNs and ENs. However, during the analysis process, the awareness of prior understanding was constantly present to minimize this risk. The analysis was validated by the authors' discussion of the findings during manuscript preparations [44].

The result of the present study shows that the night staff experiences its work as being largely invisible and unknown and not always appreciated by the day staff, although nursing care continues 24 hours a day. Many regard night work as less qualified [50] and surrounded with myths [34,51]. According to the interviewees, these myths about night work, for example, that the night staff sleeps on duty, still exist among day staff and patients. The day staff's false notions about working at night, found in this study, were also revealed in an earlier study [51]. Health care has been developing with regard to technical, medical as well as organizational matters [23,52,53], therefore the night work is increasingly beginning to resemble the day work. In this study, it was shown that the duties at night have to be performed under difficult conditions, such as working in silence with the lights dimmed, and making decisions when fatigue threatens. According to the night staff, the main goals of the work are:

• To provide the patients with rest to enable them to restore their strength for healing and treatment.

• To provide qualified care in which the RN has the medical responsibility for independent assessments.

• To put the ward in order for daytime activities and to hand it over in the best possible state.

Although the night work assignments are, according to the night staff, similar to those in the daytime, they are carried out by fewer staff. In this study, it was found that RNs and ENs appreciated their team work and relied on each other's competences and responsibilities to carry out the tasks that need to be done. This result contradicts the results from a study of nurses' roles in the daytime, where it was found that the distinction between the work roles of ENs and RNs was unclear [16].

The manning of the wards at night has increased over time at the hospital studied, despite cost savings and periodic employment freezes. However, does this mean the patients' secure and safe medical care has also increased or has the work load decreased? A previous study showed a relation between increased manning and decreased complications, as well as instances of death [54]. However, the results of this study indicate no such positive relation between more nurses and either increased care quality or decreased complications. Nevertheless, the results point to the importance of manning with regard to the patients' health status, as well as nursing and caring needs. The increased work load in combination with low manning at night could affect the nurses' experiences of their own health, which has been shown in a study about the staff's own health experiences during downsizing [26]. That study revealed that nurses working alone at night felt insecure, because they did not have any colleagues to consult in situations where they had to make assessments and decisions. According to Hertting and explicit colleagues, more substantial demands were put on the nurses at the same time as new methods of treatments were being introduced, and increasingly more advanced nursing care was being required by the patients. The results of this study indicate that work duties had been added over time, partly to occupy the night staff (RNs as well as ENs) and partly to facilitate the duties of the day staff. The higher proportion of service tasks at night has been shown in an earlier study [29]. Many of the service tasks performed at night are of the kind that should be cheaper to execute during the daytime, or by less qualified, thus less expensive, nursing staff at night. However, the night staff not only carried out service tasks for the day staff, but also prepared patients for surgery or treatment. This kind of preparation work was also included in a description of night watch activities at a post-anesthesia care unit [55].

As shown in the results, the RNs make medical assessments about whether or not to call the doctor or carry out any other treatments. This medical responsibility becomes particularly emphasized during the night when the RNs do not have a colleague close by to consult. The physician who RNs may need to consult when a patient's health status changes can be an unfamiliar doctor with whom the RN has no working relationship. A negative or intimidating relationship between a physician and a nurse has been found to be a risk factor for the safe and secure care of patients [56]. It is known that the quality of nurse-physician relationships affects the job satisfaction of nurses [57]. Does this imply that the lack of a satisfactory working relationship also means that the patients are placed in hazardous situations?

The increase in medical development and nursing demands, mentioned above, further magnifies the responsibility of having to make correct medical assessments. This additional responsibility should influence nursing education. It has been argued that a preparation program for the care of the aging population also needs to include the requirements of night shift work [58]. Consequently, the Swedish descriptions of RNs' competence [8] have to be questioned with regard to them not making any distinction between day and night work.

Nurses working at night also have the responsibility of informing new staff members, as well as judging their actual competence necessary to carry out the collaborative night work. The duties incumbent upon the RNs at night differ from the Swedish descriptions of RNs required competence [8]. Based on a holistic and ethical attitude, RNs are expected to have theoretical and practical nursing competences (nursing and caring), as well as nursing research, teaching and leadership skills. However, in this study, the night nurses' descriptions only include images of nursing and caring, and, apart from some information given to relatives, the RNs' pedagogical function is lacking in the descriptions. A previous Swedish study revealed that RNs do not provide information to patients at night [30]. Furthermore, they do not devote themselves to research, development and preventive work, or staff management. It could be argued that not using their entire range of competences might jeopardize RNs' nursing skills and cause difficulties when they change from night to daytime work. This kind of self-imposed competence limitation could be one reason for the survival of the night nursing myths.

## Conclusion

Efficient organizations require time to recover from their daily activities. In a hospital, the night is considered to be a period in which the wards are restored to order and both the day staff and the patients renew their strength to cope with the day's work and treatment. The most important point is the team work, which developed between the registered and enrolled nurses and how necessary this team work is when working at night takes the point of departure in nursing. In order for nurses working at night to be fully appreciated, the communication between day and night staff in health care organizations needs to be developed. In addition, it is important to give the night staff opportunities to use its whole field of competence developed in education and practice.

## Competing interests

The authors declare that they have no competing interests.

## Authors' contributions

KN Study design, data collection, manuscript preparation. AMC Study design, manuscript preparation. EPA Study design, analysis, manuscript preparation.

## Pre-publication history

The pre-publication history for this paper can be accessed here:



## Supplementary Material

Additional file 1 **Interview guide** Click here for file
