# Supplementary material for: Night nursing – staff's working experiences
Source: BMC Nurs. 2008 Oct 31;7:13. doi: 10.1186/1472-6955-7-13 (PMC2606673; doi:10.1186/1472-6955-7-13)
Supplement: Additional file 1 — Interview guide [file 1472-6955-7-13-S1.doc]

**Appendix**

**Night nursing**

*Interview guide*

**Theme 1**: The content of work

Initial question: Could you please tell me what the night work consists of?

Include task, work division, responsibility, demands in follow up questions if not mentioned spontaneously.

**Theme 2**: Task changes over time

Initial question: Could you please tell me, from your point of view, how the night work has changed over time?

Include changes in organization, tasks, responsibility, demands in follow up questions if not mentioned spontaneously.

**Theme 3:** The working conditions that constitute night nursing duties

Initial question: Could you please tell me what makes up night work duties?

No specific areas were prepared before the interview.
